# Remifentanil Promotes PDIA3 Expression by Activating p38MAPK to Inhibit Intestinal Ischemia/Reperfusion-Induced Oxidative and Endoplasmic Reticulum Stress

**DOI:** 10.3389/fcell.2022.818513

**Published:** 2022-01-26

**Authors:** Jiantong Shen, Yaqing Zhan, Qiulan He, Qiwen Deng, Kunhe Li, Shihong Wen, Wenqi Huang

**Affiliations:** Department of Anesthesiology, The First Affiliated Hospital, Sun Yat-sen University, Guangzhou, China

**Keywords:** remifentanil, PDIA3, p38MAPK, oxidative stress, endoplasmic reticulum stress

## Abstract

**Background:** Remifentanil protects against intestinal ischemia/reperfusion (I/R) injury; however, its exact mechanism remains to be elucidated. The objective of this study was to investigate the underlying molecular mechanism of remifentanil in intestinal I/R injury in mice.

**Methods:** We evaluated the intestine-protective effect of remifentanil in adult male mice with 45 min superior mesenteric artery occlusion followed by 4 h reperfusion by determining the following: intestinal Chiu’s scores, diamine oxidase, and intestinal fatty acid binding protein in serum; the apoptotic index, lipid peroxidation product malondialdehyde (MDA), and superoxide dismutase (SOD) activity in the intestinal mucosa; and the intestinal mRNA and protein expressions of Bip, CHOP, caspase-12, and cleaved caspase-3, reflecting endoplasmic reticulum (ER) stress. Furthermore, conditional knockout mice, in which the protein disulfide isomerase A3 (PDIA3) gene was deleted from the intestinal epithelium, and SB203580 (a selective p38MAPK inhibitor) were used to determine the role of PDIA3 and p38MAPK in I/R progression and intestinal protection by remifentanil.

**Results:** Our data showed that intestinal I/R induced obvious oxidative stress and endoplasmic reticulum stress–related cell apoptosis, as evidenced by an increase in the intestinal mucosal malondialdehyde, a decrease in the intestinal mucosal SOD, and an increase in the apoptotic index and the mRNA and protein expression of Bip, CHOP, caspase-12, and cleaved caspase-3. Remifentanil significantly improved these changes. Moreover, the deletion of intestinal epithelium PDIA3 blocked the protective effects of remifentanil. SB203580 also abolished the intestinal protection of remifentanil and downregulated the mRNA and protein expression of PDIA3.

**Conclusion:** Remifentanil appears to act *via* p38MAPK to protect the small intestine from intestinal I/R injury by its PDIA3-mediated antioxidant and anti-ER stress properties.

## Introduction

Intestinal ischemia/reperfusion (I/R) injury, a serious clinical event accompanied by high morbidity and mortality, is one of the main causes of multiple organ failure in the perioperative period (Mallick et al., 2004). Unfortunately, effective treatment strategies and drugs for intestinal I/R injury are still lacking.

Remifentanil is a potent ultrashort-acting opioid analgesic agent that is widely used as a general anesthetic (Scott and Perry, 2005; Komatsu et al., 2007). We (Shen et al., 2016) and others (Cho et al., 2013; Sayan-Ozacmak et al., 2015) have previously demonstrated that remifentanil may protect the intestine against intestinal I/R injury. However, the exact protective mechanisms of remifentanil need to be explored further.

Previously, using two-dimensional gel electrophoresis (2DE) followed by matrix-assisted laser desorption/ionization time-of-flight mass spectrometry (MALDI-TOF MS), we identified 16 differentially expressed proteins (>1.5-fold change) in the intestinal mucosa after intestinal I/R (Liu et al., 2010). Protein disulfide isomerase A3 (PDIA3, also known as ERp57, ERp60, and GRP58) is a protein that is markedly downregulated by intestinal I/R injury but upregulated by ischemia preconditioning. PDIA3 is a classic member of the protein disulfide isomerase family and mainly exists in the endoplasmic reticulum (ER) (Frickel et al., 2004). Prior research (Yoo et al., 2017) has also demonstrated that PDIA3 protein levels are significantly altered during transient spinal cord ischemia, which further was identified as a candidate therapeutic agent against spinal cord ischemic damage. Nevertheless, to date, few reports have illustrated the intestinal protective properties of PDIA3 against ischemic challenges.

ER stress can be promoted by various stressors, including hypoxia, acidosis, ATP depletion, oxidative stress, and calcium overload (Ajoolabady et al., 2021; Ren et al., 2021). Furthermore, sustained and severe ER stress leads to ER stress–related cell apoptosis, which is characterized by the upregulation of Bip, CHOP, caspase-12, and cleaved caspase-3 (Folch-Puy et al., 2016). As a multifunctional protein, PDIA3 not only plays an important role in regulating cell apoptosis induced by ER stress but also confers protective effects against oxidative stress (Coe and Michalak, 2010; Yoo et al., 2017; Yoo et al., 2019). Meanwhile, many studies (Cho et al., 2013; Zhao et al., 2013a; Qu et al., 2019; Lewinska et al., 2020; Zhou et al., 2020) have found that remifentanil reduces hypoxia/reoxygenation (H/R) or I/R-mediated oxidative stress. Previous studies (Chen et al., 2016; Chen et al., 2017) have also reported that remifentanil may protect cardiomyocytes from H/R injury by inhibiting ERS-induced apoptosis. Therefore, we explored the mechanisms of remifentanil in intestinal protection and focused on its PDIA3-mediated antioxidant and antiapoptotic properties.

p38 mitogen–activated protein kinase (p38MAPK) is a member of the MAPK family and is activated by a variety of cellular stresses, including intestinal I/R challenge (Fu et al., 2003). The p38MAPK signaling pathway is also involved in intracellular signal transduction pathways that mediate oxidative and ER stress (Imarisio et al., 2017; Han et al., 2020). Upon activation, p38MAPK is translocated to the nucleus, where it is phosphorylated and activates different transcription factors and transactivates target genes. Previously, Zhou et al. (2009) found that PDIA3 expression was decreased by PDIA3 siRNA in human breast cancer cells, but this inhibitory effect was abolished by a p38MAPK-specific inhibitor (SB203580), suggesting that p38MAPK is involved in the regulation of PDIA3 expression. However, further studies are needed to determine whether PDIA3 is regulated by p38MAPK signal transduction during intestinal protection of remifentanil against intestinal I/R injury.

Based on the aforementioned studies, we hypothesized that remifentanil could upregulate PDIA3 expression by activating p38MAPK to inhibit intestinal I/R-induced oxidative and ER stress.

## Materials and Methods

### Animals and Operative Procedures

The current study was approved by the Animal Care Committee at Sun Yat-sen University (Guangzhou, China), and the animal experimental protocols were performed in accordance with the National Institutes of Health guidelines. Adult male C57BL/6N mice (8–12 weeks old, 21–30 g) were obtained from the Animal Center of Sun Yat-sen University (Guangzhou, China). Flox mice (PDIA3 flox/flox) and conditional knockout (cKO) mice (PDIA3 flox/flox; pVillin-Cre) were purchased from Cyagen Biosciences Inc (Guangzhou, China).

All mice were anesthetized with pentobarbital (30 mg/kg, intraperitoneal injection), and the intestinal I/R model was established as described previously (Li et al., 2017a). Briefly, a 2-cm midline laparotomy was performed, and the small intestine was exteriorized. Then, the superior mesenteric artery (SMA) was identified and occluded for 45 min with an atraumatic microvascular clip, followed by removal of the clip for 4 h of reperfusion. Body temperature was maintained at 37.5 ± .5°C with warming pads during the experimental period.

### Drug Administration and Groups

Remifentanil was dissolved in 154 mM NaCl (normal saline). A selective p38MAPK inhibitor SB203580 was dissolved in dimethyl sulfoxide (DMSO), and the concentration was 40 μg/ml (Abhilasha et al., 2019). All drugs were administered *via* intraperitoneal injections (i.p.).

Experiment 1 To explore the role of PDIA3 in the intestinal protection of remifentanil.

**FIGURE 1:**
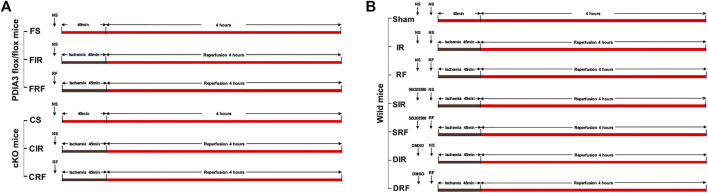
Experimental protocols. **(A)** Experiment 1. **(B)** Experiment 2. NS = normal saline; RF = remifentanil; SB203580, the selective p38MAPK inhibitor; DMSO, the solvent of SB203580.

Flox mice (PDIA3 flox/flox) were randomly assigned to FS, FIR, and FRF groups and cKO mice (PDIA3 flox/flox; pVillin-Cre) were randomly assigned to CS, CIR, and CRF groups (*n* = 5 each) as follows (Figure 1A):

1. FS: normal saline (10 ml/kg, i.p., 5 min before sham surgical preparation) + sham surgical preparation (isolation of SMA without occlusion).
2. FIR: normal saline + intestinal I/R (SMA occlusion for 45 min followed by 4 h reperfusion).
3. FRF: remifentanil (.1 μg/ml, 10 ml/kg, i.p., 5 min before I/R) + intestinal I/R.
4. CS: normal saline + sham surgical preparation.
5. CIR: normal saline + intestinal I/R.
6. CRF: remifentanil + intestinal I/R.

Experiment 2 To explore the role of p38MAPK in the intestinal protection of remifentanil.

Wild mice were randomly assigned to one of seven groups (*n* = 8 each) as follows (Figure 1B):

1. Sham: normal saline (10 ml/kg, i.p., 20 min before sham surgical preparation) + normal saline (10 ml/kg, i.p., 5 min before sham surgical preparation) + sham surgical preparation.
2. IR: normal saline + normal saline + intestinal I/R.
3. RF: normal saline + remifentanil (.1 μg/ml, 10 ml/kg, i.p., 5 min before I/R) + intestinal I/R.
4. SIR: SB203580 (selective p38MAPK inhibitor, 40 μg/ml, 10 ml/kg, i.p., 20 min before I/R) + normal saline + intestinal I/R.
5. SRF: SB203580 + remifentanil + intestinal I/R.
6. DIR: DMSO (the vehicle control of SB203580, 2%, 10 ml/kg, i.p., 20 min before I/R) + normal saline + intestinal I/R.
7. DRF: DMSO + remifentanil + intestinal I/R.

### Biological Sample Collection

At the end of the reperfusion period, blood was sampled by eyeball extirpation and centrifuged at 3,500 rpm for 15 min at 4°C, and the clear supernatants were collected for subsequent measurements of serum biochemical markers. A 10-cm segment of the ileum was harvested from the ileocecal valve and divided into two segments. The first 1 cm was fixed in 4% formaldehyde and embedded in paraffin for histological analysis and evaluation of apoptosis of the intestinal mucosal epithelium. The remaining intestinal fragment (9 cm long) was irrigated with cold normal saline. The intestinal mucosa was dried with filter paper and preserved at −80°C after being scraped off gently.

### Histological Assessments of Intestinal Injury

The segment of the small intestine was stained using hematoxylin and eosin. The stained (4 μm) sections were evaluated independently by two pathologists who were blinded to the study groups according to Chiu’s scores for the intestine, as previously described (Shen et al., 2016).

### Detection of Diamine Oxidase (DAO) and Intestinal Fatty Acid–Binding Protein (iFABP) Levels in Serum

To further assess intestinal injury, circulating DAO and iFABP levels were measured. The markers were measured using ELISA kits (Enzyme-Linked Biotechnology Co., Ltd., Shanghai, China) according to the manufacturer’s instructions. The results were expressed in pg/ml.

### Detection of Malondialdehyde (MDA) and Superoxidase Dismutase (SOD) Levels in the Intestinal Mucosa

The lipid peroxidation product, MDA, in the intestinal mucosa was measured using ELISA kits (Enzyme-Linked Biotechnology Co., Ltd., Shanghai, China). The results were expressed as nmol/g. SOD activity was evaluated by inhibition of nitroblue tetrazolium reduction by superoxide anion generated by the xanthine/xanthine oxidase system using a commercial assay kit (Enzyme-Linked Biotechnology Co., Ltd., Shanghai, China). The results were expressed as ng/g.

### Terminal Deoxynucleotidyl Transferase–Mediated dUTP–Biotin Nick-End Labeling (TUNEL) Staining

The ileal segments were fixed in 4% formaldehyde and embedded in paraffin. To assess apoptosis of the intestinal mucosal epithelial cells, the tissue sections were stained using the TUNEL method, as described previously (Shen et al., 2016). Cells with obvious dark brown staining of the nucleus and nuclear membrane were considered TUNEL-positive. Five randomly chosen observation fields were examined on each slide by two pathologists who were blinded to the study groups. The apoptotic index (TUNEL-positive cell number/total cell number × 100%) was calculated for each slide.

### Quantitative Reverse Transcription PCR (RT-qPCR) Analysis

RNA was extracted from the epithelium of the small intestine using TRIzol reagent (Invitrogen). cDNA was generated using the HiScript II Q RT SuperMix for qPCR (R222-01; Vazyme, Nanjing, China). RT-PCR analysis was performed on cDNA with the ChamQ universal SYBR qPCR Master Mix (Q711-02; Vazyme, Nanjing, China), and reactions were run on the Bio-Rad CFX96 Real-Time System (Bio-Rad, Hercules, CA). Gene expression was displayed as the fold increase, and β-actin was used as the endogenous standard.

The primer sequences used were as follows: PDIA3, forward 5′- AGA​GGC​TTG​CCC​CTG​AGT​AT-3′, reverse 5′-GCT​GAC​AAT​TCC​ATC​AGC​AGT -3′; Bip, forward 5′-TGT​CGC​CCT​CAG​ACC​AGA​A-3′, reverse 5′-GAACACA CCGACGCAGGAATA-3′; CHOP, forward 5′-GCA​GCG​ACA​GAG​CCA​GAA​TA-3′, reverse 5′-ATG​TGC​GTG​TGA​CCT​CTG​TT-3′; caspase-12, forward 5′-GACAT GCTGGATGGGGTTTT-3′, reverse 5′-CCT​CTA​CTT​TTC​TCT​TGG​ATT​CTG​A-3′; and β-actin, forward 5′-AGC​CAT​GTA​CGT​AGC​CAT​CC-3′, reverse 5′-GCTGTG GTGGTGAAGCTGTA-3′.

### Western Blot Analysis

The intestinal mucosa was harvested and suspended in lysis buffer. The insoluble cell debris was removed by centrifugation (12,000 rpm for 15 min at 4°C). The supernatants were collected, and the protein concentration of the extract was determined using a bicinchoninic acid protein assay kit (Kangcheng BioTech, Shanghai, China). The samples were then boiled for 10 min, loaded onto polyacrylamide gels for electrophoresis, and then transferred onto polyvinylidene fluoride membranes for immunoblotting. To prevent nonspecific binding, the membranes were first blocked with 5% (w/v) nonfat milk in Tris-buffered saline–Tween 20 for 1 h at room temperature and then incubated with antibodies against p-p38 (1:1,000, 4511S, Cell Signaling Technology, Danvers, United States), p38 (1:1000, 8690S, Cell Signaling Technology, Danvers, United States), PDIA3 (1:2000, ab13506, Abcam, Cambridge, United Kingdom), Bip (1:1000, 3177S, Cell Signaling Technology, Danvers, United States), CHOP (1:2500, 2895, Cell Signaling Technology, Danvers, United States), caspase-12 (1:200, sc-21747, Santa Cruz Biotechnology, Oregon, United States), cleaved caspase-3 (1:1,000, 9664S, Cell Signaling Technology, Danvers, United States), or β-actin (1:1,000, 4,970, Cell Signaling Technology, Danvers, United States) overnight at 4°C, followed by horseradish peroxidase (HRP)–labeled goat anti-mouse IgG (1:5,000, SA00001-1, Proteintech, Chicago, United States) or HRP-labeled goat anti-rabbit IgG (1:5,000, SA00001-2, Proteintech, Chicago, United States) or HRP-labeled goat anti-rat IgG (1:5,000, SA00001-15, Proteintech, Chicago, United States) for 1 h at room temperature. The blots were visualized using the LI-COR Odyssey imaging system (Shen et al., 2016) and quantified using ImageJ software (National Institutes of Health, Bethesda, MD, United States).

### Statistical Analysis

Data were presented as the mean ± standard deviation (SD) and analyzed with GraphPad Prism 8.0 software (La Jolla, CA, United States). The significance of the data was evaluated using one-way ANOVA (Tukey’s *post hoc* multiple comparison test for multiple groups). Differences were considered statistically significant at *p* < .05.

## Results

### Intestinal Mucosal Morphologic Changes

**FIGURE 2:**
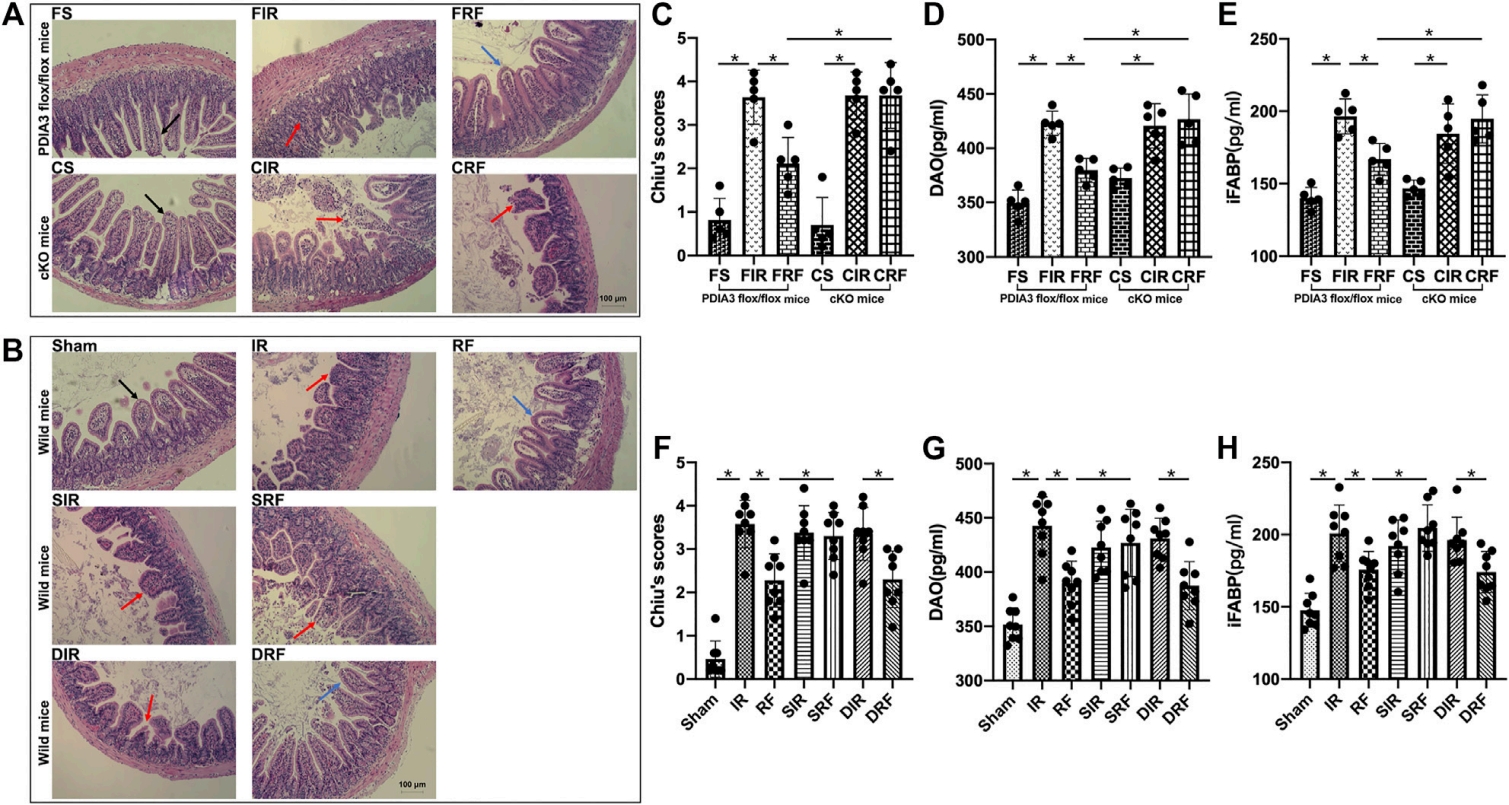
Ischemia/reperfusion injury in the intestine. Groups are the same as in Figure 1. Histopathologic changes of the intestinal mucosa were assessed by hematoxylin and eosin staining **(A,B)**, and intestinal injury was evaluated using Chiu’s scores **(C,F)** under light microscopy (×200, scale bar: 100 μm), diamine oxidase activity (DAO) **(D,G)**, and intestinal fatty acid–binding protein (iFABP) **(E,H)** in serum. In the FS, CS, and sham groups, normal intestinal villi and glands are seen and shown by black arrows. In the FIR, CIR, CRF, IR, SIR, SRF, and DIR groups, injured intestinal gland, disintegrated intestinal villi, and significantly increased gap between epithelial cells are observed and shown by red arrows, indicating severe mucosal damage. In the FRF, RF, and DRF groups, no significant edema in intestinal villi is seen, and extension of the subepithelial space (Gruenhagen space) was developed, which are shown by blue arrows, indicating slight mucosal damage. Data are expressed as mean ± SD, *n* = 5–8. The results were compared by ANOVA with Tukey’s *post hoc* test. *P < .05.

Representative mucosal morphological changes are presented in Figures 2A,B. Normal-appearing morphology with structural integrity of the villi and glands was revealed in the sham-operated mice (FS, CS, and sham groups in Figures 2A,B). In contrast, 45 min of ischemia followed by 4 h of reperfusion caused acute intestinal damage with marked edema and mucosal villi disruption, infiltration of inflammatory cells, and increased gaps between epithelial cells (FIR, CIR, and IR groups in Figures 2A,B). Mild injury to the intestinal mucosal tissues was observed in the FRF and RF groups (Figures 2A,B). Mucosal injury was quantified according to Chiu’s scores (Figures 2C,F). Chiu’s scores were significantly higher in the injury groups than those in the sham-operation group (FIR vs. FS and IR vs. sham, *p* < .05) and were attenuated by remifentanil (FRF vs. FIR and RF vs. IR, *p* < .05).

### Changes of Serum DAO and iFABP Concentration

As an index of small intestinal mucosal injury, the serum levels of DAO (Figures 2D,G) and iFABP (Figures 2E,H) in the injury groups were markedly higher than those in the sham-operation group (FIR vs. FS and IR vs. Sham, *p* < .05) and were reduced by remifentanil (FRF vs. FIR and RF vs. IR, *p* < .05).

Accompanying mucosal morphological changes, changes in Chiu’s scores and serum DAO and iFABP concentrations further indicated that remifentanil could protect the intestine against intestinal I/R injury.

### Changes in Intestinal Mucosal Epithelial Apoptosis

**FIGURE 3:**
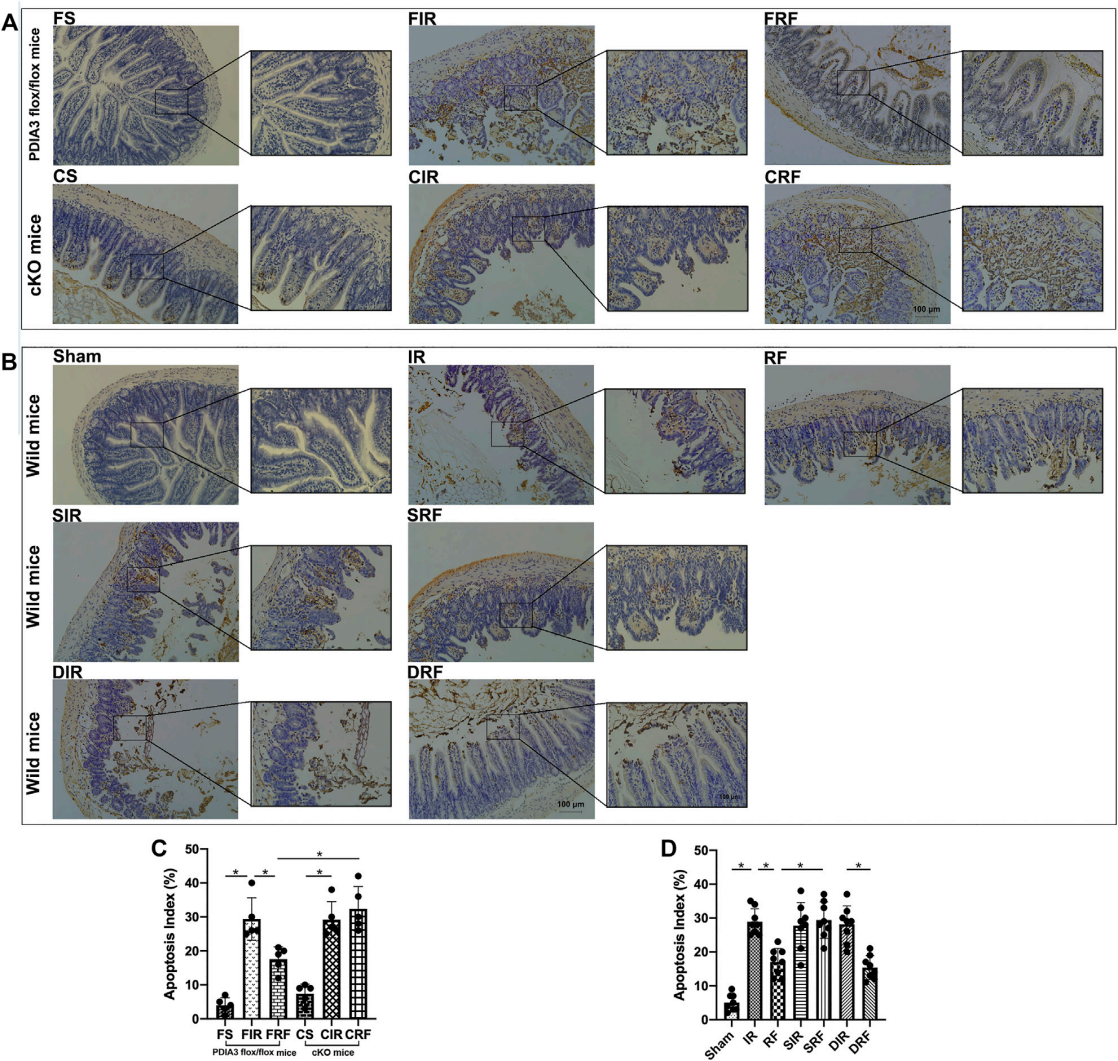
TUNEL assay of the intestinal mucosal cell apoptosis by light microscopy (×200 and ×400, scale bar: 100 μm). **(A,B)** The intestinal mucosal cell apoptosis changes assessed by TUNEL staining among the groups. Groups are the same as in Figure 1. Apoptotic nuclei are stained dark brown. In the FS, CS, and sham groups, few apoptotic epithelial cells are observed at the villous. In the FIR, CIR, CRF, IR, SIR, SRF, and DIR groups, notable destruction of the villi is evident, and apoptotic enterocytes are present in the upper regions of the villi. In addition, large numbers of detached epithelial cells with dark brown nuclei are seen in the enteric cavity. In the FRF, RF, and DRF groups, the damage of villi is slight, and there are fewer apoptotic cells than in the FIR and IR groups. **(C,D)** Apoptotic indices among the groups. Data are expressed as mean ± SD, *n* = 5–8. The results were compared by ANOVA with Tukey’s *post hoc* test. *P < .05.

As shown in Figures 3A,B, TUNEL-positive mucosal epithelial cells had nuclei that were stained dark brown. Detailed descriptions of mucosal epithelial apoptosis in different groups are showed in the legend of Figure 3. The apoptotic indices among the different groups (Figures 3C,D) were significantly higher in the injury model groups than those in the sham-operation groups (FIR vs. FS and IR vs. Sham, *p* < .05). Administration of remifentanil markedly decreased the apoptotic index (FRF vs. FIR and RF vs. IR, *p* < .05).

### Changes in Intestinal Mucosa–Relevant mRNA Expressions

**FIGURE 4:**
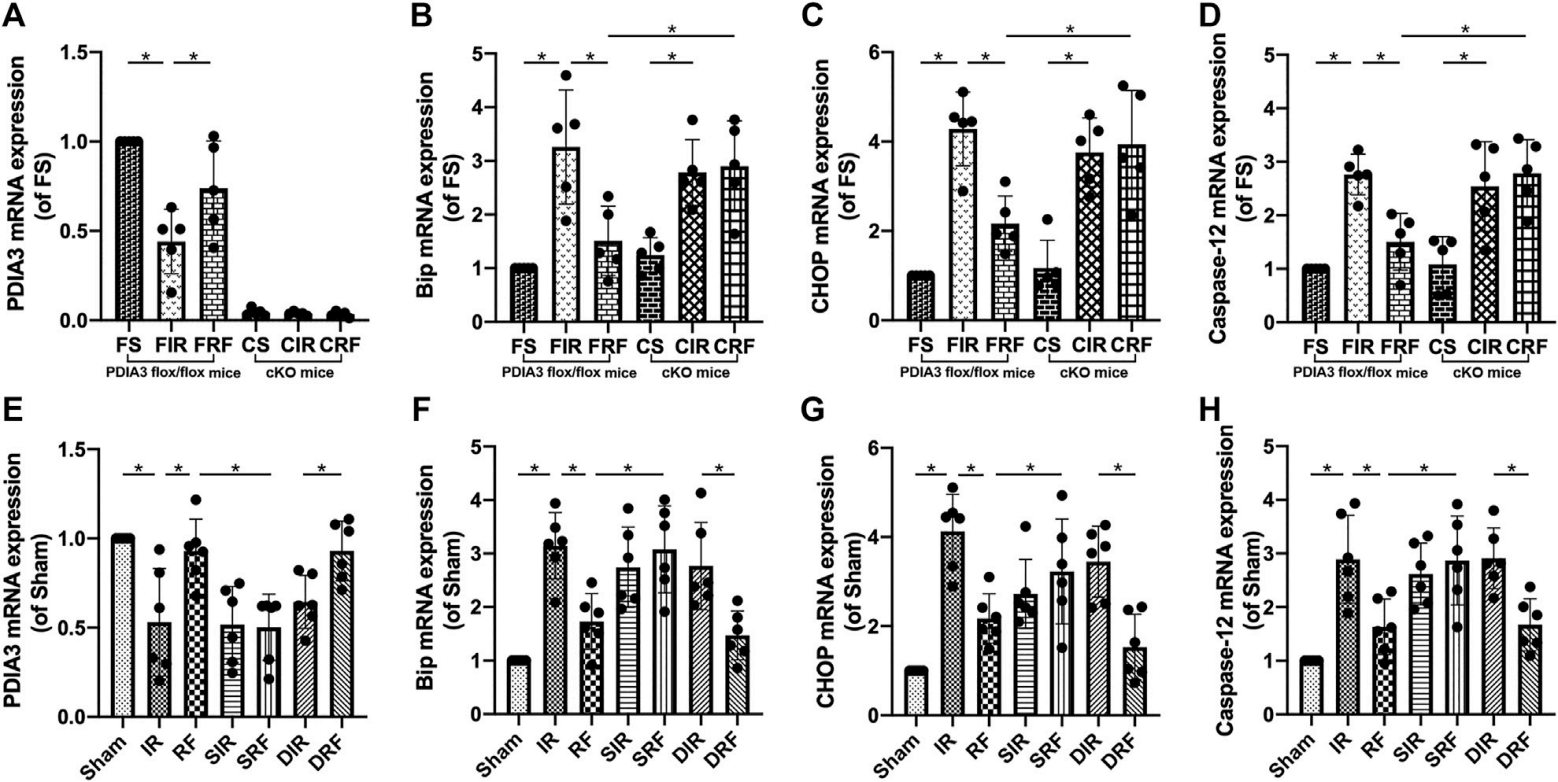
Changes in intestinal mRNA expression of the relevant proteins. Groups are the same as in Figure 1. The changes in PDIA3, Bip, CHOP, and caspase-12 mRNA levels in the intestine are shown in **(A–H)** (*n* = 5–6). Data are expressed as mean ± SD. The results were compared by ANOVA with Tukey’s *post hoc* test. *P < .05.

As shown in Figures 4B–D,F–H, after 4 h of reperfusion, the mRNA levels of the ER stress–related genes Bip, CHOP, and caspase-12 were significantly upregulated, indicating enhanced ER stress in the intestinal tissues (FIR vs. FS and IR vs. Sham, *p* < .05). These mRNA levels were markedly reduced by remifentanil (FRF vs. FIR and RF vs. IR, *p* < .05).

### Changes in Intestinal Mucosa–Relevant Protein Expressions

**FIGURE 5:**
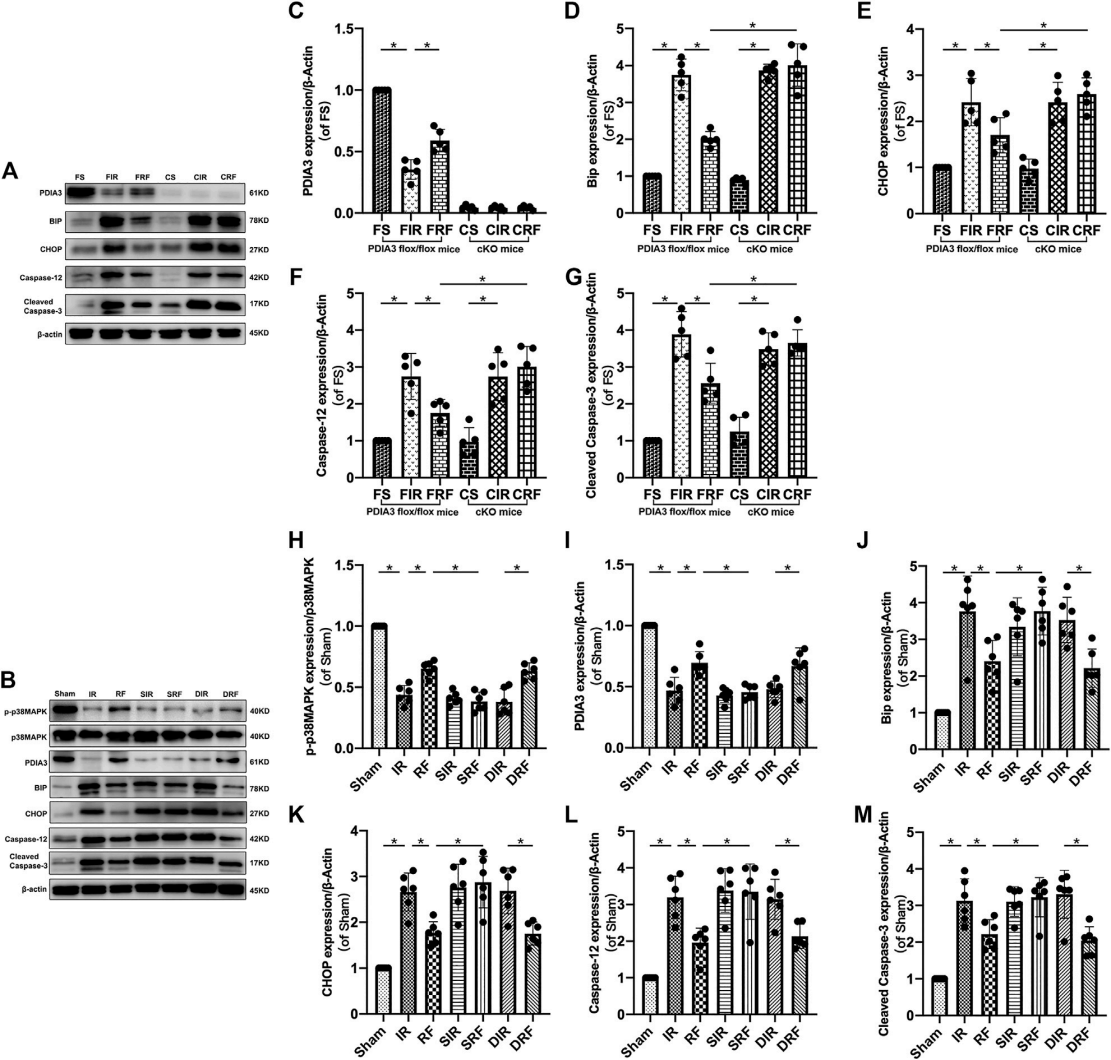
Changes in relevant intestinal protein expression. Groups are the same as in Figure 1. Representative Western blot bands **(A,B)** show p-p38MAPK, p38MAPK, PDIA3, Bip, CHOP, caspase-12, cleaved caspase-3 and β-actin expression in the intestinal mucosa. Quantitative changes in protein concentrations **(C–M)** are shown by densitometry analysis from the Western blotting assay (*n* = 5–6). Data are expressed as mean ± SD. The results were compared by ANOVA with Tukey’s *post hoc* test. *P < .05.

Representative Western blot bands of relevant proteins in the intestinal mucosa are shown in Figures 5A,B. Quantitative changes in protein concentrations (Figures 5C–M) are shown by densitometry analysis from Western blotting assay. Compared to the sham-operation group, there was a notable upregulation in the protein expression of Bip, CHOP, caspase-12, and cleaved caspase-3 after I/R injury (FIR vs. FS and IR vs. Sham, *p* < .05). In addition, remifentanil significantly decreased the expressions of these proteins (FRF vs. FIR and RF vs. IR, *p* < .05).

Accompanying the changes in intestinal mucosal epithelial apoptosis (Figure 3) and mRNA (Bip, CHOP, and caspase-12) expressions (Figure 4), the changes in protein (Bip, CHOP, caspase-12, and cleaved caspase-3) expressions (Figure 5) further indicated that remifentanil protected the intestine against intestinal I/R injury by inhibiting ER stress–related cell apoptosis.

### Changes in Intestinal Mucosal MDA and SOD Concentration

**FIGURE 6:**
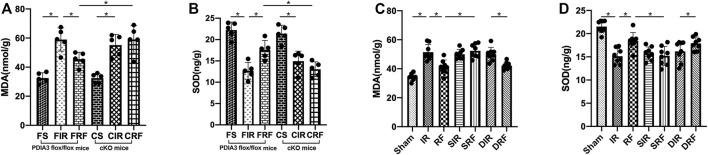
Changes in intestinal mucosa malondialdehyde (MDA) **(A,C)** and superoxide dismutase (SOD) **(B,D)**. Groups are the same as in Figure 1. Data are expressed as mean ± SD, *n* = 5–8. The results were compared by ANOVA with Tukey’s *post hoc* test. *P < .05.

As shown in Figures 6A,C, MDA levels in the intestinal mucosa after I/R injury were significantly increased (FIR vs. FS and IR vs. sham, *p* < .05). Conversely, the SOD activity obviously decreased as shown in Figures 6B,D (FIR vs. FS and IR vs. sham, *p* < .05). In addition, remifentanil significantly reduced MDA levels and notably enhanced SOD levels in the intestinal mucosa (FRF vs. FIR and RF vs. IR, *p* < .05). These changes in intestinal mucosal MDA and SOD concentrations demonstrated that remifentanil could alleviate oxidative stress induced by intestinal I/R.

### The Role of PDIA3 in the Intestinal Protection of Remifentanil

As shown in Figures 2C–E, Chiu’s scores and serum DAO and iFABP concentrations in the CRF group were similar to those in the FIR and CIR groups (CRF vs. FIR and CRF vs. CIR, *p* > .05) and significantly higher than those in the FRF group (CRF vs. FRF, *p* < .05), indicating that the deletion of PDIA3 from intestinal mucosa completely abrogated the intestinal protective effect of remifentanil.

As shown in Figures 3C, 4B–D–D, 5D–G–G, the apoptotic index and mRNA (Bip, CHOP, and caspase-12) and protein (Bip, CHOP, caspase-12, and cleaved caspase-3) expressions in the CRF group were markedly higher than those in the FRF group (CRF vs. FRF, *p* < .05), and similar to those in the FIR and CIR groups (CRF vs. FIR and CRF vs. CIR, *p* > .05), indicating that remifentanil upregulated PDIA3 expression to protect against intestinal I/R injury *via* inhibition of ER stress–related cell apoptosis.

As shown in Figures 6A,B, the intestinal mucosal MDA and SOD concentrations in the CRF group were similar to those in the FIR and CIR groups (CRF vs. FIR and CRF vs. CIR, *p* > .05). There was an obvious increase in intestinal mucosal MDA and a marked decrease in the intestinal mucosal SOD in the CRF group compared to those in the FRF group (CRF vs. FRF, *p* < .05), suggesting that the antioxidant stress property of PDIA3 contributed to the intestinal protection of remifentanil.

Meanwhile, there were no statistical differences in the abovementioned variables in comparison between the FS and CS groups and between the FIR and CIR groups (FS vs. CS and FIR vs. CIR, *p* > .05).

### The Role of p38MAPK in the Intestinal Protection of Remifentanil

As showed in Figures 2F–H–H, 3D, 4F–H–H, 5J–MJ–M5J–M, 6C–D–D, there was an obvious increase in Chiu’s scores, serum DAO and iFABP concentration, apoptotic index, mRNA (Bip, CHOP, and caspase-12) protein (Bip, CHOP, caspase-12, and cleaved caspase-3) expressions, and intestinal mucosal MDA concentration, with a marked decrease in intestinal mucosal SOD, in the SRF group compared with those in the RF and DRF groups (SRF vs. RF and SRF vs. DRF, *p* < .05). These variables in the SRF group were similar to those in the IR, SIR, and DIR groups (SRF vs. IR, SRF vs. SIR, and SRF vs. DIR, *p* > .05). These results indicate that pretreatment with SB203580 (a selective p38MAPK inhibitor) abolished the protective effects of remifentanil on intestinal I/R injury.

As shown in Figures 4, 5, the p-p38MAPK protein expression and the mRNA and protein expression of PDIA3 in the intestinal mucosa were significantly lower in the SRF than those in the RF and DRF groups (SRF vs. RF and SRF vs. DRF, *p* < .05), and similar to those in the IR, SIR, and DIR groups (SRF vs. IR, SRF vs. SIR, and SRF vs. DIR, *p* > .05), suggesting that activation of p38MAPK may upregulate PDIA3 expression, contributing to the protective effect of remifentanil.

Meanwhile, SB203580 or DMSO alone had no effect on the abovementioned variables in the comparison between the SIR or DIR group and IR group, or between the DRF and RF groups (SIR vs. IR, DIR vs. IR, and DRF vs. RF, *p* > .05).

## Discussion

This study presents three important findings. First, a single dose of remifentanil (1 μg/kg) administered intraperitoneally before intestinal ischemia protected the intestine from I/R injury by reducing oxidative and ER stress. Second, we generated pVillin-Cre–mediated PDIA3 cKO mice (PDIA3 flox/flox; pVillin-Cre), in which the PDIA3 gene was deleted specifically in the intestinal epithelium, and demonstrated that PDIA3 with antioxidant and anti-ER stress properties played a key role in the intestinal protection of remifentanil. Third, after applying a selective p38MAPK inhibitor (SB203580), remifentanil appeared to protect against intestinal I/R injury *via* p38MAPK to enhance PDIA3 expression.

Several studies have provided evidence that remifentanil can mediate protective effects against intestinal I/R injury. One study (Sayan-Ozacmak et al., 2015) showed that pretreatment with remifentanil can remarkably attenuate the intestinal I/R injury, possibly by lowering lipid peroxidation and leukocyte infiltration. Another study (Cho et al., 2013) demonstrated that a single bolus of remifentanil inhibited local oxidative stress and systemic inflammation and protected against I/R injury in the small intestine. We (Shen et al., 2016) previously found that remifentanil preconditioning attenuated both intestinal I/R injury *in vivo* and IEC-6 cell OGD/R injury *in vitro* by inhibiting intestinal mucosal epithelial cell apoptosis *via* δ- and μ-opioid receptors but not δ-opioid receptors. A growing body of literature has shown that both oxidative and ER stress contribute significantly to epithelial damage during intestinal I/R injury (Bi et al., 2020; Ding et al., 2020; Hartmann et al., 2017; Liu et al., 2019). As a result, oxidative stress in the ER caused by intestinal I/R accelerates the accumulation of unfolded and misfolded proteins, ultimately leading to apoptosis (Jiang et al., 2020; Yin et al., 2020; Yoo et al., 2019). Consistently, in the current study, we found that remifentanil significantly suppressed intestinal I/R-induced oxidative stress, as evidenced by decreased intestinal mucosal MDA and increased intestinal mucosal SOD levels, as well as downregulated ER stress, as evidenced by the decreased mRNA and protein expressions of Bip, CHOP, caspase-12, and cleaved caspase-3 in the intestine.

PDIA3 facilitates isomerization of the disulfide bond in nascent and denatured proteins with a thioredoxin-like domain, thereby playing an important role in ER stress (Hatahet and Ruddock, 2009; Kozlov et al., 2010). In the present study, our results showed that PDIA3 was upregulated after remifentanil administration, which blocked the ER stress–associated apoptotic pathway, as evidenced by a distinctly elevated expression of Bip, CHOP, caspase-12, and cleaved caspase-3 in the intestinal epithelium. These results are mostly consistent with those of a previous study (Yoo et al., 2019) which demonstrated that exogenous PDIA3 protein (Tat-PDIA3) could protect against ischemic damage, likely by attenuating ER stress–induced apoptosis through CHOP. As expected, our results showed that remifentanil significantly promoted PDIA3 upregulation to decrease the lipid peroxidation product MDA while restoring SOD activity in the intestinal mucosa. The antioxidative effect of PDIA3 in the current study concurred with the findings of (Yoo et al., 2017; Yoo et al., 2019), who found that PDIA3 attenuated oxidative damage by ameliorating ischemia-induced lipid peroxidation and reducing antioxidant enzymes.

Growing evidence demonstrates that the p38MAPK family plays an important role in intracellular signal transduction in response to extracellular stimuli, including intestinal I/R challenge (Fu, Xing, et al., 2003; Fu, Yang, et al., 2003; Guo et al., 2016; Li et al., 2021; Li, Xu, et al., 2017; Liu et al., 2020; Ming et al., 2019; Murayama et al., 2006; Santén et al., 2009; Yusof et al., 2009; Zheng et al., 2005). Dexmedetomidine (Liu et al., 2020), propofol (Li et al., 2021), and 6-gingerol (Li, Xu, et al., 2017) have been shown to protect against intestinal I/R injury *via* p38MAPK inhibition, wherein p38MAPK activation conferred detrimental effects on intestinal I/R progression. However, in our current study, phosphorylation of p38MAPK was significantly downregulated after 45 min of ischemia followed by 4 h of reperfusion but was obviously enhanced by remifentanil treatment, as confirmed by Western blot analysis. Moreover, pretreatment with the p38MAPK-specific inhibitor SB203580 abolished p38MAPK activation and reversed the intestinal protective effects of remifentanil. This finding is partly consistent with that of previous studies. Previous studies (Zhao, Liu, et al., 2013; Zhu et al., 2014) found that hypoxic postconditioning mediated neuroprotection against transient global cerebral ischemia in rats and mice by increasing the phosphorylation of p38MAPK, which may be attributable to mitochondrial translocation of Bcl-2–related protein Bcl-xL. Another study (Guan et al., 2014) provided evidence that cerebral ischemic preconditioning induces ischemic tolerance by activating the p38MAPK signaling pathway in the rat brain. Taken together, these results suggest that remifentanil-induced p38MAPK signaling pathway activation plays an important role in mitigating intestinal I/R injury.

Controversy continues with respect to the functions of p38MAPK phosphorylation. We speculate that these two factors might have contributed to this. First, the duration of ischemia and/or reperfusion varies, meaning that samples from different time points were collected, leading to different levels of phosphorylated p38MAPK. Second, different subtypes of p38MAPK, such as p38α, β, δ, and γ, with different or even opposite functions (Beenstock et al., 2014; Grossi et al., 2014), may be activated at different time points during the progression of I/R.

A previous study (Zhou et al., 2009) found that PDIA3 expression was decreased by PDIA3 siRNA in human breast cancer cells, and this inhibitory effect was abolished by a p38MAPK-specific inhibitor (SB203580), suggesting that the expression of PDIA3 requires the p38 MAPK pathway. In contrast, our study found that activation of p38MAPK promoted PDIA3 expression, as evidenced by the notable downregulation of the mRNA and protein expression of PDIA3 after p38MAPK-specific inhibitor administration. The relationship between p38MAPK and PDIA3 is an interesting area for future studies.

Our study had several limitations. First, it was not designed to determine the effects of remifentanil on long-term mouse survival and gut function, nor was the relationship between opioid receptor activation and p38MAPK/PDIA3 upregulation investigated. Also, only one dose of remifentanil was used to investigate its intestinal protective effects and explore the relevant mechanism. Nevertheless, more doses should be used to further investigate the dose–effect relationship. In addition, no *in vitro* experiment has been performed to further confirm the ER stress–associated mechanism of remifentanil against intestinal I/R injury. Therefore, more *in vitro* studies potentially using RNA interference technology may help elucidate this.

## Conclusion

Collectively, our study provides evidence that remifentanil promotes PDIA3 expression by activating p38MAPK to inhibit intestinal I/R-induced oxidative and ER stress. PDIA3, a therapeutic candidate protein, may be a new target for the prevention and treatment of intestinal injury after intestinal I/R.

## Data Availability

The original contributions presented in the study are included in the article/Supplementary Material; further inquiries can be directed to the corresponding authors.
